# Creation of Individual Scientific Concept-Centered Semantic Maps Based on Automated Text-Mining Analysis of PubMed

**DOI:** 10.1155/2018/4625394

**Published:** 2018-07-26

**Authors:** Ekaterina Ilgisonis, Andrey Lisitsa, Valerya Kudryavtseva, Elena Ponomarenko

**Affiliations:** ^1^Institute of Biomedical Chemistry, RAS, Moscow, Russia; ^2^KuB Ltd., Russia

## Abstract

Concept-centered semantic maps were created based on a text-mining analysis of PubMed using the BiblioEngine_v2018 software. The objects (“concepts”) of a semantic map can be MeSH-terms or other terms (names of proteins, diseases, chemical compounds, etc.) structured in the form of controlled vocabularies. The edges between the two objects were automatically calculated based on the index of semantic similarity, which is proportional to the number of publications related to both objects simultaneously. On the one hand, an individual semantic map created based on the already published papers allows us to trace scientific inquiry. On the other hand, a prospective analysis based on the study of PubMed search history enables us to determine the possible directions for future research.

## 1. Introduction

Today, the number of papers and citations is considered as the main indicator of scientific output [1]. According to the PubMed/MEDLINE database [2], the number of scientific articles in the fields of medicine and biology amounted to more than a million over the last year. To analyze the knowledge accumulated in the form of hundreds and even thousands of papers, a large number of text-mining solutions, often called the “PubMed derivatives”, have been proposed [3].

MeSH indexing terms (the main headings and subheadings) are served as a rich resource for extracting a broad range of domain knowledge [4]. In 2015, it was shown that leveraging the implicit and explicit semantics provided by manually assigned MeSH-terms is an effective representation for capturing the underlying context of complex associations [5]. Earlier researches in this field including the development of a MeSH-based index of research interests confirmed MeSH's usefulness for indexing research interests [6], development of a biomedical expert finding system [7], or creation of an individualized service on the library's portal system [8]. As a visualization technique, many tools help to organize the PubMed publications in the form of a concept-centered network or graph [3, 9–11].

In this study, we developed an idea of combined MeSH-based profiles of the published and recently viewed papers in the form of a MeSH-centered semantic network. Past research in this field including an analysis of MeSH indexing patterns consists of Unified Medical Language System [12] (UMLS) semantic groups related to the MeSH headings together with their associated MeSH subheadings [13], which suggests that the MeSH-based analytical tools tend to be more effective over time.

An example of the use of automated text processing is an approach implemented in BiblioEngine_v2018 software and based on the analysis of the summary of scientific publications on medical and biological topics from the PubMed/MEDLINE database. Automated analysis and comparison of MeSH-terms included in the papers enable us to form groups of relevant literature according to user-defined criteria and highlight the key concepts within these groups expressed in the form of a relationship between MeSH. This approach was implemented in the automated analysis of scientific papers in the field of molecular mechanisms of the onset of Alzheimer's disease, investigation of drug transport systems, and discovery of natural compounds with therapeutic properties [14].

In this work, concept-centered semantic maps were created based on a text-mining analysis of PubMed using the BiblioEngine_v2018 software. The edges between the two objects (*«*concepts*»*) were automatically calculated based on the index of semantic similarity, which is proportional to the number of publications related to both objects simultaneously. On the one hand, an individual semantic map created based on the already published papers allows us to trace scientific thought. On the other hand, a prospective analysis based on the study of search queries to PubMed enables us to determine the possible directions for future research.

The proposed approach was demonstrated using an example of the creation of an individual cognitive map of Professor Alexander Archakov, a biochemist and one of the most famous Russian scientists in the field of life science. Archakov A.I. is a founder of a scientific school in the field of molecular organization studies. His interests are focused on the study of functioning the oxygenase cytochrome P450-containing system, molecular mechanisms, structure, and function of membranes and biological oxidation. Being the pioneer in the development of proteomics in Russia, Archakov A.I. headed the Chromosome 18 Team in the international Human Proteome Project [15]. The construction of the scientist's semantic map is an interesting example since his scientific interests are changed every 10-15 years covering biochemistry, bioinformatics, nanotechnology, proteomics, personalized medicine, and digital health.

## 2. Materials and Methods

To download a list of relevant literature published in PubMed, we used the query [“*Archakov A.I.*”]. A list of identifiers of relevant publications in PubMed (PMIDs) was created, and a list of MeSH associated with each article was loaded (see Supplementary Note 1 for algorithm details).

To quantify the semantic relationships between the nodes of the semantic map (MeSH-terms here), the BiblioEngine software was used [11]. The closest nodes on the semantic map were determined as the terms most frequently occurred in the same papers. Selection of MeSH, specific to the articles published by Alexander Archakov, was performed by choosing MeSH, the frequency of occurrence of which was significantly different from that in the random sample. Our random sample consisted of a list of PMIDs randomly assembled from all PMIDs presented in the PubMed/MEDLINE library, and the size of the group that contained those randomly selected PMIDs was equal to the number of PMIDs presented in the targeted group, in this case, the number of PMIDs of Archakov's scientific articles.

To assess how the scientific interests have changed over time, all the papers published were divided into groups according to the release date (starting from the year 1970). Thus, we have formed five groups including the articles published in 1970-1980, 1980-1990, 1990-2000, 2000-2010, and 2010-2017, respectively. The time intervals were chosen in such a way that, on the one hand, the selections of papers were comparable in volume, and, on the other, they covered a period that was significant for the research. For each time interval, a list of PMIDs and the corresponding MeSH was formed.

The semantic concept-centered map was created based on the calculated matrix of semantic similarity between the nodes (MeSH-terms) and then visualized in the Cytoscape v.3.6.1 program [16]. The coefficient of semantic similarity T (*a*, *b*) between two objects (MeSH* a* and MeSH* b*) was calculated using the Tanimoto normalization [17]:

(1) $$\begin{array}{c} T\left(\;a,b\;\right)=\frac{\left|P_{ab}\right|}{\left|P_{a}\right|+\left|P_{b}\right|-\left|P_{ab}\right|}; \end{array}$$

where* Pa* is the number of PMIDs of the papers indexed with MeSH of* a*, obtained as the number of papers returned by PubMed to a query (“*a*” [MeSH-Terms]”),* Pb* is the number of PMIDs of papers indexed with MeSH of* b*, and* Pab* is the number of PMIDs of papers indexed with MeSH of* a, *as well as MeSH of* b*.

The nodes formed a separate cluster if the measure of semantic similarity between the nodes was in the range of 0.75 to 1. For each time interval, the MeSH-centered clusters were visualized as a semantic network. Additional information for annotation of the semantic network was obtained from GoPubMed system [18] (http://gopubmed.org) and the Scopus bibliometric database (https://www.elsevier.com/solutions/scopus). We used GoPubMed for creating a list of journals, in which relevant research articles retrieved from PubMed were published, and information on the number of citations was obtained from Scopus.

For the creation of the MeSH-centered semantic network, based on search history, BioKnol [14] software was used. The free available plugin that enables us to trace the whole search history in the form of PMIDs or DOI of the content can be installed from http://ws.bioknowledgecenter.ru. While navigating PubMed, a researcher generates user-specific reading profiles that can be shared within the BioKnol social networking environment. Within the framework of the present study, we used the browsing history of scientific articles in the PubMed system for a one-year period (2012-2013). Reflecting Archakov's present interests, this period also allows us to compare MeSH with his scientific papers published in 2014-2017.

## 3. Results and Discussion

Over the period from 1968 to 2017, more than 600 publications authored by Alexander Archakov were published, of which 424 papers are indexed in the PubMed system. This number is sufficient enough for demonstrating the «*proof-of-concept*». Indeed, the number of objects on the semantic map and its informativeness depend on the number of published articles. Our previous experience [19] has shown that one hundred publications are the quantity that allows us to compare the results of text-mining processing with those of biocuration and expert analysis.

The most cited articles (data about citing obtained from Scopus) are published in such journals as Proteomics, Biosensors & Bioelectronics, Journal of Proteome Research, Biochemical and Biophysical Research Communications and Biochemistry and Molecular Biology International, according to GoPubMed annotations.

**Figure 1:**
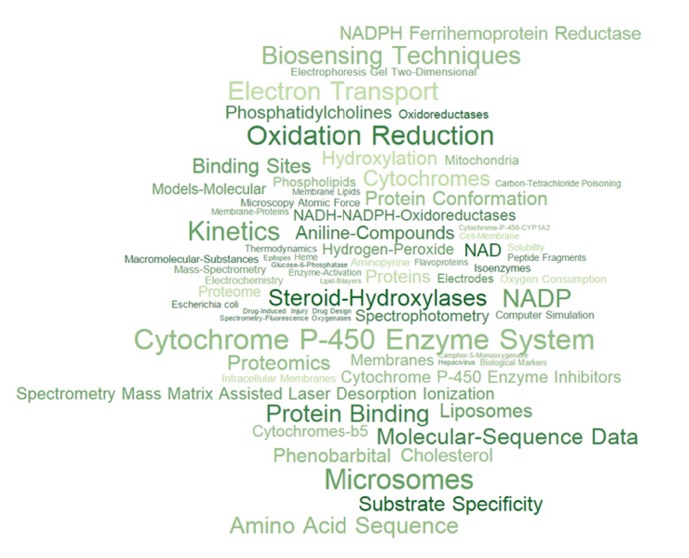
The cloud of MeSH-terms associated with the publications of Alexander Archakov in PubMed. The frequency of MeSH occurrence in the articles published is proportional to the font size.

The subjects of all published articles were presented in the form of a MeSH-terms cloud (see Figure 1), while the frequency of MeSH occurrence is proportional to the font size. The key terms are Oxidation Reduction and Cytochrome P450 Enzyme System since the main publications are focused on the study of the functional role of enzymes of the cytochromes P450 superfamily. The reaction of monooxygenase catalysis involving the participation of these proteins is a necessary link in providing vital activity; therefore, a significant part of the work is devoted to the analysis of the substrate specificity (“*Substrate Specificity*”) of proteins of this group and investigation of the kinetics of enzymatic reactions (“*Kinetics*”). Among the commonly used methods of research are two-dimensional electrophoresis (“*Electrophoresis, Gel, Two-Dimensional*”), spectrophotometric methods (“*Spectrophotometry*”), biosensor analysis (“*Biosensing Techniques*”), and mass spectrometric methods (including MALDI “*Spectrometry, Mass, Matrix-Assisted Laser Desorption-Ionization*”). Bioinformatic approaches are represented by algorithms for the analysis of amino acid sequences of proteins (“Molecular Sequence Data”), reflecting some articles led by Alexander Archakov and aimed at studying the approaches enabled us to classify proteins of the cytochromes P450 superfamily [20, 21].

**Figure 2:**
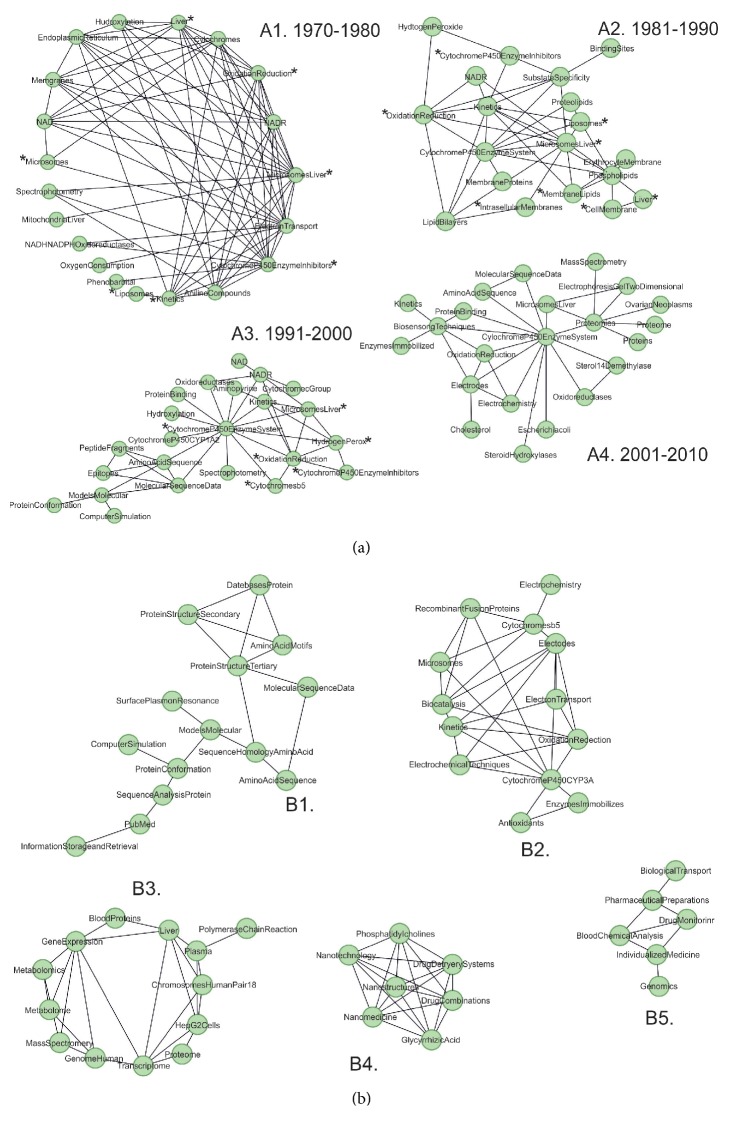
A fragment of the cognitive map created based on the analysis of scientific papers of Professor Alexander Archakov. The nodes are MeSH-terms associated with the papers. The edges between the two objects were automatically calculated based on the index of semantic similarity, which is proportional to the number of publications related to both objects simultaneously. (a) The period from 1970 to 2010. The scientific papers of each decade are presented on A1-A4 fragments. The MeSH-terms common to the A1-A4 fragments are marked with (*∗*). (b) The period from 2010 to 2017.

For a retrospective analysis of changes in the priorities of the scientific school founded by Alexander Archakov, we analyzed the frequency of MeSH-terms occurrence for the scientific papers published between 1970 and 2010 (see Figure 2(a), Supporting information, Table 1). A1-A4 fragments reflect a network of MeSH-terms associated with publications of each decade. It is noteworthy that for a quite large time interval—forty years—the key list of the most common MeSH remained almost unchanged: “*Microsomes, Liver*”, “*Cytochrome P450 Enzyme System*”, “*Oxidation-Reduction*”, “*NADH, NADPH Oxidoreductases*”, and other terms (marked in the figure with (*∗*)) characterizing the research in the field of microsomal oxidation and the molecular organization and functioning of cytochrome P450 containing system.

During the first decade under consideration (1970-1980), the specificity of the work performed is related to the study of biological membranes and electron transport, which is specified by the presence of terms such as “*Membranes*” and “*Electron transport*” on the A1 fragment. After 1980, “*Liposomes*,” “*Cholesterol*,” “*Rabbits*,” “*Phospholipids*,” and “*Membrane lipids*” are found among MeSH-terms that indicates the beginning of work on the mechanisms of damage and effective recovery of biological membranes. In the future, publications in this area will become the basis for the creation of a hepatoprotector «Phosphogliv». One of the main components of this medicine, soybean phosphatidylcholine (lecithin), can restore the structure of the liver cells, acting as a “membrane glue” and “gluing”, the defects of damaged biological membranes, regardless of their origin [22]. Today, «Phosphogliv» is a medication that can be bought from any pharmacy. Therefore, the description of these publications is only present in a retrospective part of the semantic map.

The last decade of the previous century (since 1991) signaled the beginning of active bioinformatics research on the structure and function of proteins of the cytochromes P450 superfamily. This period is specified by the presence of decade-specific MeSH-terms such as “*Amino Acid Sequence*,” “*Molecular Sequence Data*,” “*Molecular Models*,” “*Binding sites*,” “*Computer Simulation*,” and “*Protein Conformation*” (see the A3 fragment of Figure 2). The beginning of the 21st century (2000-2010) is associated with the development of postgenomic experimental methods, as can be seen from the MeSH most commonly found in this period, “*Proteomics*,” “*Mass-Spectrometry*,” and “*Electrophoresis Gel Two-dimensional*.” Also, for the first time, there are publications associated with the terms “*Microscopy, Atomic Force*” and “*Electrochemistry*” (A4 fragment, Figure 2), which indicate a new period of research.

After 2010, more than 40 articles led by Alexander Archakov were published, the main MeSH-terms of which are presented in Figure 2(b). As part of the analyzed fragment of the cognitive map, we can identify five clusters that characterize the main areas of research carried out by the team of the Institute of Biomedical Chemistry headed by Professor Alexander Archakov since 1989.

Thus, the first cluster (B1) reflects studies in the field of bioinformatics, the development of information systems for storage of proteomic data (“*Databases Protein*”), algorithms for sequence analysis (“*Sequence Analysis Protein*”) and molecular modeling (“*Models molecular*”), and research in the field of text-mining analysis (“*PubMed*”, “*Information Storage and Retrieval*”). The second fragment (B2) reveals the scientific interests of biochemical laboratories, the continuation of research in the field of cytochrome P450-containing and enzymatic systems. A group of MeSH-terms describing bioelectrochemistry studies stands out (see MeSH “*Electrochemistry*,” “*Electrodes*,” and “*Electrochemical Techniques*”). Members of the team headed by Professor Victoria Shumyantseva, working in this field, are the leaders recognized by the world community [23–25].

The department of proteomic research was established in 2001 and has increased significantly over the past ten years when Russia took part in the international Human Proteome Project [26]. MeSH characterizing the main scientific papers in the field of proteomics are presented in the B3 fragment of Figure 2, “*Blood Proteins*” (the main biological material for this research is the depleted plasma of human blood [27] and “*Chromosome Pair 18*” (chromosome 18 was selected for research within the Russian part of the international Human Proteome Project [28]). The terms “*Transcriptome and Metabolome*” emphasize the need to use a system approach at the interface of several technologies in modern scientific research.

The work performed under the leadership of Alexander Archakov resulted in an increased knowledge in the field of molecular functioning of living systems that is necessary for improving the methods of diagnosis and treatment of diseases and potentially cost-effective for the use in medical practice. Thus, the other two fragments, B4 and B5, are connected with personalized medicine. The B4 fragment specifies nanotechnology studies, namely, the development of new drugs (see MeSH “*Drug Delivery Systems*”, “*Drug combination*”) and the use of nanotechnology for creating a basis in the field of analytical technologies with the potential for disease diagnostics (“*Nanomedicine*”, “*Microscopy, *and* Atomic Force*”). Studies in personalized medicine are reflected by MeSH forming the B5 fragment of the cognitive map. They are devoted to the use of sets of genomic data and postgenomic technologies for monitoring human health, including blood composition, development of the approaches to individualized drug therapy, and prediction of the risks of diseases.

Citation is one of the knowledge-intensive indicators that indirectly enables us to assess the relevance of published work. It is believed that citation in rapidly developing scientific fields is higher than in others. When analyzing the paper activity and bibliometric indicators, the citation statistics of the works published stands out; according to Google Scholar (https://scholar.google.ru) as of January 2017, the articles of Alexander Archakov were cited about 13 thousand times, while the* h-*index of the scientist was 50.

The maximum number of citations (2552) was received by the book «Lipid peroxidation in biological membranes» coauthored by Yuriy A. Vladimirov and published in 1972; the volume “Microsomal Oxidation” (1975) is cited in more than three hundred scientific works, and the book “Cytochrome P-450 and active oxygen” published in 1990 ranks third with respect to citations among books in the field of biochemistry published by Alexander Archakov.

**Figure 3:**
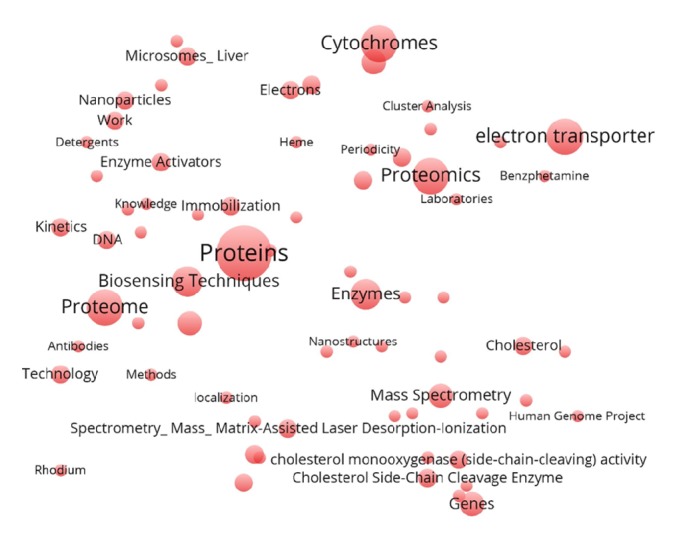
MeSH-terms associated with the 20 most cited publications of Alexander Archakov. The frequency of MeSH occurrence is proportional to the font size. The distance between the nodes is random.

The most cited papers in journals are related to proteomics and participation in the international projects [29–32]; mostly these are the articles published between 2003 and 2011. An analysis of MeSH-terms associated with the 20 most cited articles of Alexander Archakov showed the importance of these proteomic studies for the scientific community (see Figure 3), characterized by MeSH-terms “*Proteins*”, “*Proteome*”, “*Mass-spectrometry*”, etc., as well as fundamental biochemical papers presented in the figure with the key terms “*Enzyme Activators*”, “*Enzymes*”, “*Electron transporter*”, etc. It is noteworthy that the results of the analysis reflect the basic information about the nature of the scientific work of the scientist, previously obtained from the entire array of published papers. It seems that MeSH networks represent information comparable with a citation graph [33]. The most cited publications (at least in the example described) thematically coincide with the most frequently encountered MeSH-terms characterizing the entire sample of the articles published by the author, i.e., the subject, to which the most attention was devoted.

The scientist's cognitive map constructed based on the* published* studies was supplemented with information about the key MeSH associated with the articles* viewed* by the scientist. For the analyzed period (2012-2013), the BioKnol [14] automatic system recorded a log with 114 different scientific papers published in international journals and indexed by PubMed/MEDLINE. For identifying common concepts, the frequencies of MeSH-terms were compared with the key words of the articles published under the guidance of Alexander Archakov during the period 2014-2017.

**Figure 4:**
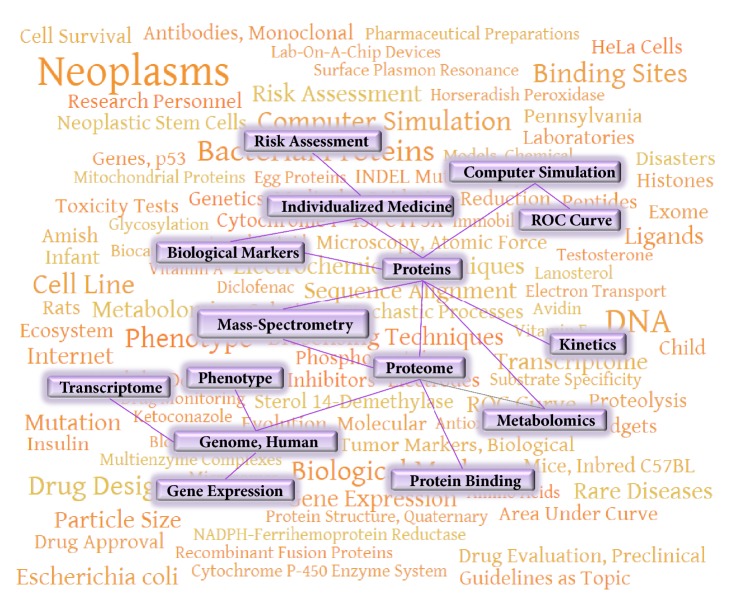
A fragment of the cognitive map constructed based on an analysis of the frequencies of MeSH-terms for the scientific papers viewed by the scientist in 2012-2013. In the foreground, there is a fragment of the MeSH network associated both with the articles viewed and with the author's articles published in the latest years (2014-2017).

Therefore, two sources of MeSH-terms were considered. On one hand, there were frequencies of MeSH-terms, which occur in the articles published that are written by the author (or coauthors). On the other hand, there were frequencies of MeSH-terms, which characterize scientific papers read by the same author. The difference between MeSH profiles of written/published and read papers is useful to capture the new trends in the personal research interests. For instance, for professor Archakov's sample for the period from 2014 to 2017 terms “Proteomics”, “Enzyme Activation”, and “Membrane Proteins” significantly prevailed in connection with the published articles, as compared to the articles read. This observation reflects the field of the scientific interests and the process of knowledge actualization in this area (see Figure 4, the background cloud of MeSH).

MeSH-terms appeared more frequently in published papers include terms “*Individualized medicine*,” “*Risk Assessment*,” “*Biological markers*,” “*Protein interaction mapping*,” etc. It is interesting that an analysis of MeSH-terms of the articles published in 2014-2017 (i.e., for the subsequent period) shows the appearance of the same terms in the scientist's publications describing the possibilities of postgenomic methods for personalized medicine. The key studies published in this field in the last two years [34, 35] highlight the possibilities of metabolic profiling for diagnosis. The studies [36, 37] reveal the possibilities of using proteomic methods and mass-spectrometry for medicine and a search for biological markers. The studies [38, 39] discuss the expression of potentially medically important genes at the proteomic level based on the analysis of genomic and transcriptomic data.

Figure 4 demonstrates a fragment of the cognitive map showing MeSH-terms, the frequencies of which have significantly increased in the scientist's articles published in 2014-2017. Thus, the development of this direction, namely, an automatic processing of the history of the researcher's log to scientific papers, may be potentially interesting for prediction of possible directions for future research, the author's scientific publications, and, consequently, the effectiveness of his scientific activity. However, besides BioKnol, there are alternative ways to store the search history. The key is to compare the frequencies of MeSH-terms associated with already published studies and MeSH-terms assigned with the content of the scientist log-history formed during routine work.

From our point of view, the retrospective part of an individual semantic map is less subject to temporal changes. This mainly depends on the age of a researcher: the younger the scientist is, the more the changes on the retrospective map can be expected including radical changes in its structure like the emergence of a new cluster of terms if a researcher has changed his place of work or a field of study. The prospective part of a map is more susceptible to changes since it depends on the cognitive interest of a scientist. The higher the cognitive activity is, the more active a person is and the greater the number of scientific directions (and consequently MeSH-terms) can be reflected in a personal semantic map. The predictive power of a MeSH-centered semantic map depends on the level of the nodes hierarchy, MeSH-terms. The more detailed (or less) the level of the MeSH-terms hierarchy on a prospective semantic map is, the more precise one can assume the specificity of scientific papers.

As an example of the BiblioEngine software capabilities, we shall consider the creation of a semantic map in the field of scientific developments of John Craig Venter (JC Venter), who had a significant impact on the development of postgenomic technologies and molecular biology. John Craig Venter is a biologist, businessman, and a cofounder of the Institute for Genomic Research and J.* Craig Venter Institute*, which conducts research in synthetic biology.

**Figure 5:**
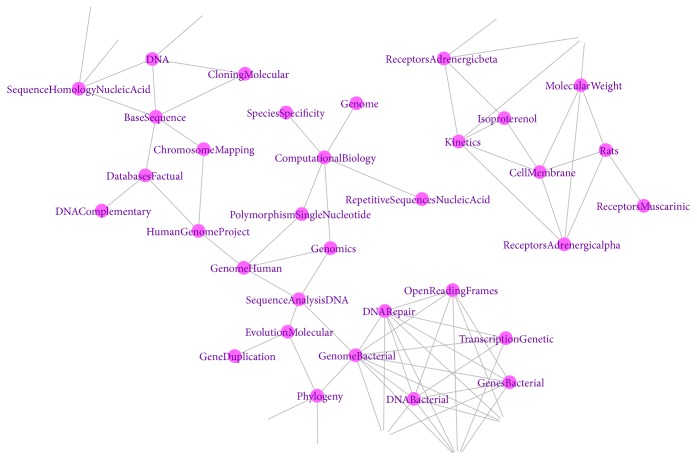
A fragment of the MeSH-centered semantic map created based on the analysis of John Craig Venter's publications. The nodes are MeSH-terms associated with the articles.

Figure 5 shows a fragment of his semantic map. The larger subgraph contains the terms “*Genomics*,” “*Human Genome Project*,” “*Sequence Analysis DNA*,” and others, which highly correspond to one of the activities of Craig Venter, i.e., participation in the project of sequencing the human genome. Some of the concepts included in the same subgraph (for example, “*Genome Bacterial*,” “*Recombination Genetics*”) refer to the activities of Craig Venter, which he carries out in the field of synthetic biology.

Another subgraph consisted of the terms “*Receptors*,” “*Isoproterenol*,” and others (see Figure 5). This group of terms characterizes the field of scientific interests of Craig Venter related to the study of alpha- and beta-adrenergic receptors, immobilized drugs, and their influence on the cardiac muscle. In PubMed, 25 publications were available on the query “*Venter JC catecholamines*.” All these publications date back to the period 1975-1990. They describe the properties of immobilized catecholamines. Another search query, “*Venter JC isoproterenol*,” also revealed a similar number of publications that coincide with the publications found for “*Venter JC catecholamines*” query. Thus, the BiblioEngine enabled, in a few hours, obtaining a semantic map reflecting the main concepts of the sphere of Craig Venter's scientific interests, as well as a list of his most important publications in each area.

**Figure 6:**
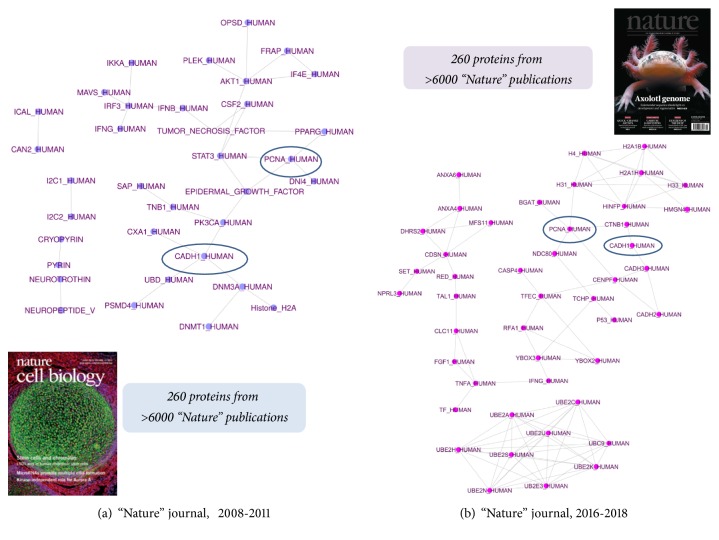
Protein-centered semantic map created based on the analysis of the papers from “Nature” journal: (a) 2008-2011 years; (b) 2016-2018 years.

The approach suggested can also be illustrated by constructing the semantic networks for proteins mentioned in the articles published in* Nature*. We have chosen 260 most commonly discussed proteins in two time periods 2008-2011 and 2016-2018. For each group of proteins, we analyzed the frequency of protein occurrence in all published in the PubMed database articles in the selected period. We visualized the most strong associations in the networks in Figure 6, which illustrates associations between the most popular proteins in 2008-2011 years. In the center of the network, there are cancer-associated proteins, such as “*Tumor necrosis factor*” and “*Epidermal Growth factor*”. From Figure 6(b) it is clear that after 10 years the focus of scientific interest has shifted to* ubiquitin*. Ubiquitinylation of proteins of some signaling pathways regulates their activity and, as a result, mediates signal transmission to the nucleus. Finally, as it has been recently discovered, the functions of ubiquitin are also applied to the regulation of the nucleus apparatus: its role in regulating the transcription of genes by modifying the RNA polymerase complex [40]. A comparison of the two protein lists showed that they have two common proteins:* PCNA_HUMAN* and* CADH1_HUMAN*.

The simple idea of using BiblioEngine and BioKnol allows users to form concept-centered semantic networks (maps), organizing real-time PubMed-available knowledge in the form of semantic networks. Networks represent relationships between various objects: genes (proteins), MeSH, chemical compounds, names, terms from the dictionary compiled by experts, names for providing information of an individual or group scientific output, etc.

Semantic networks were visualized using Cytoscape according to the matrix of similarity, and the distance between the nodes (concepts) was correlated with the normalized number of popular scientific articles. Relevant fragments of a network, as well as a list of PMIDs for each relationship detected, could be provided to an expert for the future analysis.

## 4. Conclusions

This work shows the possibility of using the BiblioEngine software package combined with the BioKnol social network for an automated text-mining analysis of scientific literature and creation of a personal cognitive map. By the example of scientific publications of Alexander Archakov, a semantic map of key MeSH-based concepts was constructed, allowing us to trace the main scientific directions of his work. The practical use of the data obtained is directly related to the possibility of predicting the scientific output of an individual or a group of researchers. One of the proposed methodical solutions is to use the results of a text-analysis of the articles viewed since this indicator is one of the most important factors in the scientific search of a scientist. This study demonstrates an obvious correlation between viewed, already published scientific articles, and those that will be published in the future. Approved methodological approaches can be applied to other authors and represent practical significance in terms of developing modern approaches to evaluation of scientific output.

## Data Availability

The data used to support the findings of this study are available from the corresponding author upon request.

## Supplementary Materials

Supplementary 1 Supplementary Table 1: evolution of the scientific interests of Alexander Archakov over the past 50 years.

Supplementary 2 Supplementary Note 1: BiblioEngine Toolkit Manual.
