# Supplementary material for: Creation of Individual Scientific Concept-Centered Semantic Maps Based on Automated Text-Mining Analysis of PubMed
Source: Adv Bioinformatics. 2018 Jul 26;2018:4625394. doi: 10.1155/2018/4625394 (PMC6083525; doi:10.1155/2018/4625394)
Supplement: Supplementary 1 — Supplementary Table 1: evolution of the scientific interests of Alexander Archakov over the past 50 years. [file 4625394.f1.docx]

|  | *YEARS* | | | | |
| --- | --- | --- | --- | --- | --- |
| *MeSH-term* | *1970-1980* | *1981-1990* | *1991-2000* | *2001-2010* | *2010-2017* |
| Cytochrome P-450 Enzyme System | 29 | 31 | 39 | 32 | 9 |
| Oxidation-Reduction | 23 | 19 | 16 | 15 | 4 |
| Microsomes, Liver | 37 | 30 | 18 | 12 | 0 |
| Kinetics | 17 | 20 | 16 | 9 | 4 |
| Liver | 23 | 5 | 4 | 4 | 2 |
| NADP | 19 | 5 | 8 | 0 | 0 |
| Membranes | 18 | 0 | 0 | 0 | 0 |
| NAD | 16 | 1 | 3 | 0 | 0 |
| Cytochromes | 26 | 1 | 0 | 0 | 0 |
| Electron Transport | 24 | 2 | 3 | 4 | 3 |
| Amino Acid Sequence | 0 | 3 | 11 | 13 | 3 |
| Hydrogen Peroxide | 0 | 6 | 12 | 0 | 0 |
| NADPH-Ferrihemoprotein Reductase | 0 | 4 | 12 | 4 | 4 |
| Substrate Specificity | 0 | 9 | 8 | 2 | 5 |
| Cholesterol | 3 | 11 | 6 | 4 | 0 |
| Liposomes | 6 | 11 | 6 | 1 | 0 |
| Protein Conformation | 0 | 9 | 5 | 3 | 1 |
| Membrane Lipids | 1 | 8 | 1 | 0 | 0 |
| Phospholipids | 5 | 8 | 2 | 1 |  |
| Molecular Sequence Data | 0 | 3 | 11 | 11 | 4 |
| Steroid Hydroxylases | 0 | 0 | 25 | 7 | 0 |
| Binding Sites | 3 | 3 | 9 | 9 | 2 |
| Proteomics | 0 | 0 | 0 | 21 | 5 |
| Spectrometry, Mass, Matrix-Assisted Laser Desorption-Ionization | 0 | 0 | 0 | 17 | 3 |
| Biosensing Techniques | 0 | 0 | 5 | 25 | 10 |
| Proteome | 0 | 0 | 1 | 9 | 7 |
| Biological Markers | 0 | 0 | 0 | 4 | 6 |
| Protein Binding | 5 | 2 | 7 | 11 | 7 |
| Thermodynamics | 0 | 3 | 2 | 3 | 5 |
| Electrophoresis, Gel, Two-Dimensional | 0 | 0 | 1 | 11 | 1 |
| Electrochemical Techniques | 0 | 0 | 0 | 0 | 7 |
| Microscopy, Atomic Force | 0 | 0 | 1 | 7 | 5 |
| Mass Spectrometry | 0 | 0 | 1 | 6 | 5 |

**Table 1. Evolution of the scientific interests of Alexander Archakov over the past 50 years.** MeSH-terms among the TOP-10 MeSH for this time interval are highlighted in grey. MeSH-terms not included in the TOP-10 but covering a certain number of papers in this time interval are highlighted in red. MeSH-terms, for which there are no papers in this time interval, are highlighted in green.
