# Supplementary material for: Creation of Individual Scientific Concept-Centered Semantic Maps Based on Automated Text-Mining Analysis of PubMed
Source: Adv Bioinformatics. 2018 Jul 26;2018:4625394. doi: 10.1155/2018/4625394 (PMC6083525; doi:10.1155/2018/4625394)
Supplement: Supplementary 2 — Supplementary Note 1: BiblioEngine Toolkit Manual. [file 4625394.f2.docx]

106 lines

BiblioEngine Toolkit .2018: Text-mining tool for highlighting concepts from PubMed

2018-04-06

Authors: Ekaterina Ilgisonis, Andrey Lisitsa, Valeria Kudryavtseva, Elena Ponomarenko

******************************Project Description************************************

Project Description:

For a given PubMed query, the BiblioEngine Toolkit .2018 pipeline retrieves relevant papers, extracts different types of objects - MeSH terms (gene/protein names, compounds, diseases, authors names etc.). The BiblioEngine Toolkit .2018 builds semantic relationships between objects (nodes), while the edges are proportional to the number of publications in which objects are co-occurred.

*********************************Dependencies****************************************

Dependencies:

The tool is implemented in the programming language Perl 5.10, the module for loading data from the Medline/PubMed library is implemented using Entrez Programming Utilities. During the work of the software complex, the following external sources are accessed via the Internet: the Medline/PubMed library (download of publications); the UniProt knowledge base (export of gene and protein names); database PubChem (export of names of chemical compounds).

Demo-version of the scripts are accessible at [http://195.178.207.138/projects/ !obolochka/start.py](http://195.178.207.138/projects/%20!obolochka/start.py) (please, use ​​login: exactus1, ​​pass: biblioengine)

*********************************Required Files**************************************

Required Files:

*******Scripts:

Create_keys_list_shell.pl - creating a list of keywords using as PubMed query, added by user

Load_keys_ids_shell.pl – downloading PMIDs relevant to the list of keywords from PubMed. Use $data_Keys_list.csv as input file.

Load_mesh_shell.pl – downloading the MeSH terms for the PMIDs list. Use $data_Ids_list.csv as input file.

Count_mesh_shell.pl - calculating the frequency of occurrence of loaded MeSH. Use $data_Mesh_terms.csv as input file.

Get_random_ids_shell.pl – randomly generating a list of PMIDs. Use the number of PMIDs which are needed to generate (added by user) and file million.txt

Infotable_shell.pl – select specific for target group MeSh terms by comparison frequence of co-occurrence in Target and Random group. Use $data_Sort_mesh.csv and $data_Rand_sort_mesh.csv as input files. Ratio of co-occurrence frequencies entered by user.

Load_relevant_shell.pl - download relevant papers (PMIDs) for $data_Names.csv. Use $data_Names.csv as input file. Input parameters: limit on the maximum number of PMIDs for one term (default = 50), years (default= 1900-2010).

Load_related_shell.pl - upload related publications (PMIDs) for PMIDs from $data_List_of_relevant.csv.

Matrix_shell.pl - Calculation of the matrix of relations between objects (MeSH terms). Use $data_Relevant.csv and $data_Related.csv as input files.

Subgraphs_shell.pl -selecting object and clusters for visualization. Input parameters (default - cutoff: 0.05, minsize: 3).

*******Text Files:

Million.txt - Millions of randomly selected PMIDs (example)

***********************************Output Files**************************************

*In the specified main folder:

$data_Keys_list.csv – list of keywords

$data_Ids_list.csv – list of PMIDs of relevant papers retrieved from PubMed (*Target* group)

$data_Mesh_terms.csv – list of MeSH terms for the PMIDs list.

$data_Sort_mesh.csv – list of MeSH terms with the number of times that this term was occur in the $data_Mesh_terms.csv

$data_Random_ids.csv - list of randomly selected PMIDs from PubMed (*Random* group)

$data_Rand_mesh_terms.csv - list of MeSH terms with the number of times that this term was occur in the $data_Random_ids.csv

$data_Rand_sort_mesh.csv - list of MeSH terms with the number of times that this term was occur in the $data_Rand_mesh_terms.csv

$data_Names.csv – list of specific for target group MeSH terms for which semantic network will be created

$data_Relevant.csv – list of relevant papers (PMIDs) associated with each term from $data_Names.csv

$data_List_of_relevant.csv - list of unique PMIDs for terms from $data_Names.csv

$data_Related.csv – list of related papers (PMIDs) associated with $data_List_of_relevant.csv

$data_List_of_related.csv - list of unique PMIDs for terms from $data_Related.csv

$data_Mtx_relevant.csv – list of connected objects, included the weight of the edges between nodes and PMIDs describe the relationship

$data-subgraphs-0.05-3.csv – list of objects associated with the cluster. File contain name of the cluster, number of objects, and list of the objects.

$data-subgraphs-0.05-3.tab - list of objects expected to be visualized using CytoScape (input file for Cytoscape).

**************************************Instructions*************************************

Instructions:

*** create a list of keywords using as PubMed query by running Create_keys_list_shell.pl. User need to set a list of keywords.

*** download relevant to the list of keywords PMIDs from PubMed by running Load_keys_ids_shell.pl. Use $data_Keys_list.csv as input file.

*** download the MeSH terms for the PMIDs list by running Load_mesh_shell.pl. Use $data_Ids_list.csv as input file.

*** calculate the frequency of occurrence of loaded MeSH by running Count_mesh_shell.pl

*** Randomly generating a list of PMIDs (control/random group) by running Get_random_ids_shell.pl. Use the number of PMIDs which are needed to generate (added by user) and file million.txt

*** download the MeSH terms for the PMIDs list by running Load_mesh_shell.pl. Use $data_Random_ids.csv as input file.

*** calculate the frequency of occurrence of loaded MeSH by running Count_mesh_shell.pl Use $data_Rand_mesh_terms.csv as input file.

*** Select specific for target group MeSH terms by running Infotable_shell.pl. Use $data_Sort_mesh.csv and $data_Rand_sort_mesh.csv as input files. Ratio of co-occurrence frequencies entered by user.

*** download relevant papers (PMIDs) for $data_Names.csv by running Load_relevant_shell.pl. Use $data_Names.csv as input file. Input parameters: limit on the maximum number of PMIDs for one term (default = 50), years (default= 1900-2010).

*** (optionally) upload related publications (PMIDs) for PMIDs from $data_List_of_relevant.csv by running Load_related_shell.pl.

*** Calculation of the matrix of relations between objects by running Matrix_shell.pl.

*** Selecting object and clusters for visualization by running Subgraphs_shell.pl. Input parameters (default - cutoff: 0.05, minsize: 3).

Download and install the visualization Cytoscape program from <http://www.cytoscape.org/download.php>

Use $data-subgraphs-0.05-3.tab as input file for Cytoscape and semantic ntwork visualization.

All necessary files are available at https://www.dropbox.com/sh/p8moc38amvotu31/AACt0F7fboTGSY9n-_jFMlhsa?dl=0
